# Complete genome sequence and plasmid of *Staphylococcus xylosus* isolated from milking parlor equipment at a dairy farm

**DOI:** 10.1128/mra.00899-26

**Published:** 2026-09-24

**Authors:** Emma Hinchliffe, Samuel Zavislan-Pullaro, Diego Manriquez, Elizabeth Hemming-Schroeder, Zaid Abdo, Carolina Mehaffy

**Affiliations:** 1 Department of Microbiology, Immunology, and Pathology, Colorado State University 164597, Fort Collins, Colorado, USA; 2 AgNext, Department of Animal Sciences, Colorado State University 728642, Fort Collins, Colorado, USA; Wellesley College, Wellesley, Massachusetts, USA

**Keywords:** genome, plasmid, mastitis, antimicrobial resistance

## Abstract

The study reports the genome of a *Staphylococcus xylosus* strain isolated from a recently used teat cup liner in a dairy farm. The genome length of this *S. xylosus* isolate was 2.87 Mb with 2,614 CDS. The annotated genome included putative antimicrobial resistance genes.

## ANNOUNCEMENT

Bovine mastitis from bacterial infection is one of the most common causes of disease in dairy cattle (1). In addition to causing disease in cattle, mastitis can lead to abnormal or decreased milk production. *Staphylococcus xylosus* has been identified as a cause of bovine mastitis and is known for being particularly difficult to treat due to its ability to form biofilms, which reduces the efficacy of antibiotics (2). The organism in this study was sampled in September 2025 from recently used milking parlor equipment at a local dairy farm in Northern Colorado, USA. The sample was collected by swabbing within the teat cup liner, then streaked on mannitol salt agar (MSA) and incubated at 37°C for 48 h. A singular mannitol-fermenting colony was selected and streaked for isolation on a new MSA plate and incubated at 37°C for 24 h. Following isolation, the sample was analyzed using MALDI-ToF and was determined to be *S. xylosus* with a match score of 2.40 (high-confidence identification). Moreover, a Gram stain indicated that this isolate was a gram-positive *Staphylococcus*.

The isolate was incubated in LB medium at 22°C for 24 h to prepare for DNA isolation. DNA isolation was performed using the Monarch HMW DNA Extraction Kit for Tissue (NEB), using the gram-positive protocol. An ONT library was prepared using the SQK-RBK114-24 kit and sequenced for 72 h on a GridION using flow cell FLO-MIN114 (ONT) at the Institute of Arctic Biology’s Genomics Core Lab. Reads were called and filtered (>Q10) using Dorado v7.9.8 and the base-calling algorithm dna_r10.4.1_e8.2_400bps_sup@v5.0.0 (3). Sequencing resulted in 76,312 raw reads with an *N*_50_ of 20,987 bp, as calculated by NanoPlot v0.2.4 (4). Before DNA reads were assembled, the raw reads were filtered using Porechop v0.2.4 (5) and Filtlong v0.2.1 (6) to remove adapters and reads shorter than 1 kbp. The reads were assembled with Flye v2.9.6 (7). The final consensus sequence was polished using four rounds of Racon (8) and Minimap (9) and finalized utilizing Medaka (10). Final annotation was performed using the PGAP v6.1 tool in NCBI (11–13), resulting in 2,614 CDS and 2,727 genes, including 81 RNA genes. All genome assembly, quality control, and polishing were performed using the Galaxy Platform (14, 15), with default parameters used for all tools unless otherwise specified.

**Fig 1:**
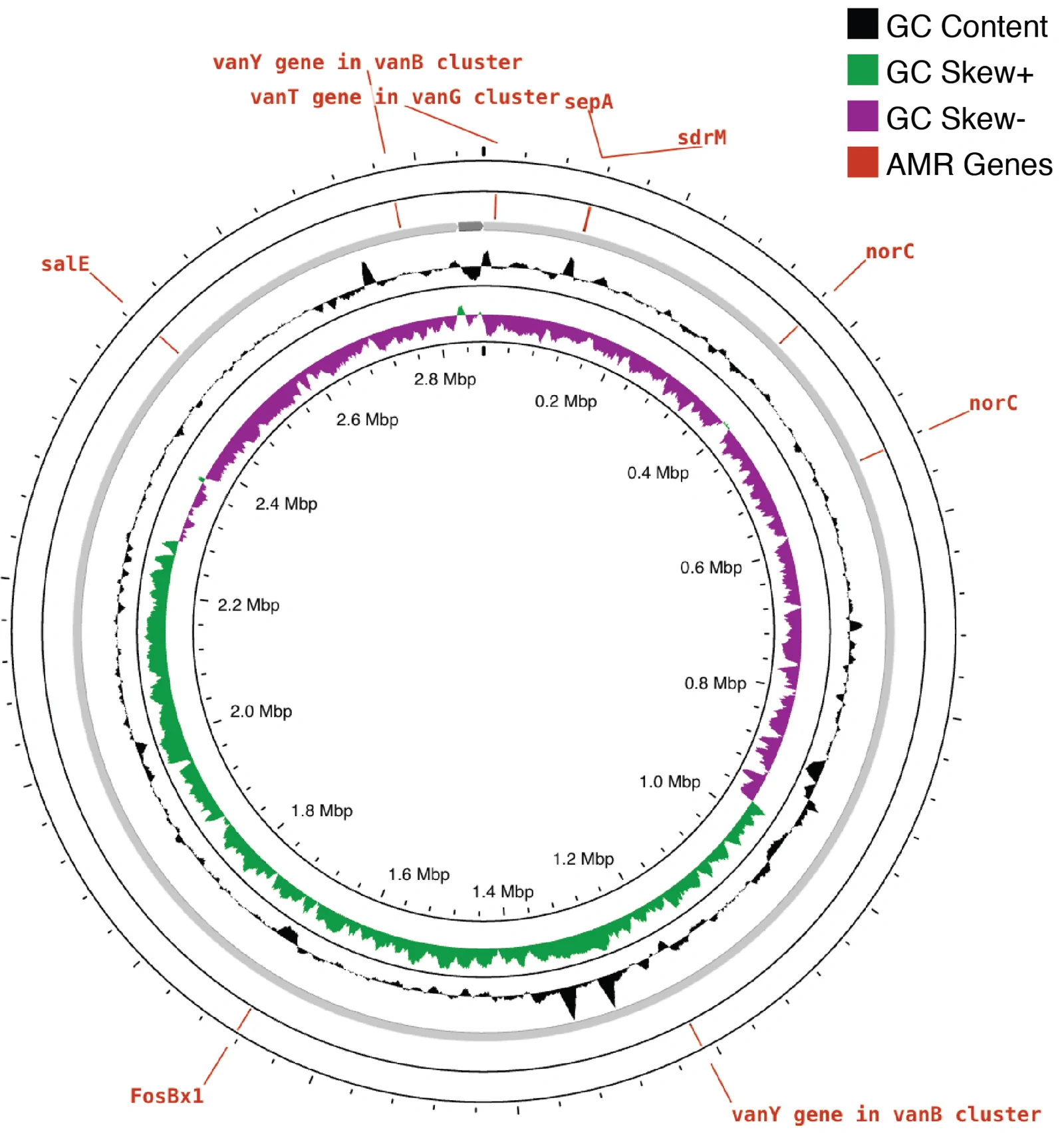
Map of genome after polishing, identifying AMR genes, GC content, and GC skew. All AMR genes were found on the chromosome, not on the plasmid.

After polishing, Quast v5.3.0 (16) was run on the genome and determined that it had 32.81% GC content, which is consistent with the typical GC content for *S. xylosus*. The assembled genome of this isolate was 2,865,445 bp in length, with mean coverage per contig of 271. Specifically, assembly resulted in two contigs, one large circular chromosome (2,837,243 bp; coverage, 270×) and one circular plasmid (28,202 bp; coverage, 278×) unique to the organism. Circularity was determined by Flye. The genome was not rotated. The polished consensus sequence and antimicrobial resistance (AMR) genes were visualized in Proksee (17) and annotated with the CARD Resistance Gene Identifier v6.0.5 (CARD DB v4.0.1) (18) (Fig. 1). Multiple putative efflux pump genes were identified in this isolate, as well as other putative resistance genes against disinfectants and cleaning products (Table 1).

**TABLE 1:** Putative AMR genes including target compunds and resistance mechanisms identified within the isolate genome^*a*^

Putative antimicrobial resistance gene	Target compound	Resistance mechanism	Reference
*salE*	Lincosamides and class A streptogramins	Antibiotic target protection, prevents binding	19
*vanY* gene in *vanB* cluster	Glycopeptide antibiotic	Antibiotic target alteration	20
*vanY* gene in *vanG* cluster	Glycopeptide antibiotic	Antibiotic target alteration	21
*sepA*	Disinfecting agents, dyes, antiseptics	Small multidrug resistance antibiotic efflux pump	22
*sdrM*	Fluoroquinolone antibiotic, disinfecting agents, antiseptics	Major facilitator superfamily (MFS) antibiotic efflux pump	23
*norC*	Fluoroquinolone antibiotic, disinfecting agents, antiseptics	MFS antibiotic efflux pump	24
*FosBx1*	Fosfomycin, phosphonic acid antibiotic	Antibiotic inactivation	25

^*a*^ All information was identified using CARD.

## Data Availability

This Whole-Genome Shotgun project has been deposited in DDBJ/ENA/GenBank under the accession number PRJNA1366496 and BioSample SAMN53832847. Raw reads can be accessed with accession number SRR36530835, and assembly can be accessed under accession number ASM5411296v1. The version described in this paper is the first version.
